# CCL2 promotes macrophages-associated chemoresistance via MCPIP1 dual catalytic activities in multiple myeloma

**DOI:** 10.1038/s41419-019-2012-4

**Published:** 2019-10-14

**Authors:** Ruyi Xu, Yi Li, Haimeng Yan, Enfan Zhang, Xi Huang, Qingxiao Chen, Jing Chen, Jianwei Qu, Yang Liu, Jingsong He, Qing Yi, Zhen Cai

**Affiliations:** 1 Bone Marrow Transplantation Center, The First Affiliated Hospital, School of Medicine, Zhejiang University, Hangzhou, China; 2 Center for Hematologic Malignancy, Research Institute, Houston Methodist, Houston, TX USA

**Keywords:** Cancer microenvironment, Chemokines, Myeloma

## Abstract

We previously showed that the chemokine CCL2 can recruit macrophages (Mφs) to the bone marrow (BM) in multiple myeloma (MM) and that myeloma-associated Mφs are important in drug resistance. Here, we explore the role of increased CCL2 expression in the BM microenvironment of MM and elucidate the underlying mechanism. Our results show that CCL2 expression is associated with the treatment status of MM patients. Mφs interact with MM cells and further upregulate their expression of CCL2. These increased level of CCL2 polarizes Mφs toward the M2-like phenotype and promotes Mφs to protect MM cells from drug-induced apoptosis. Mechanistically, CCL2 upregulated the expression of the immunosuppressive molecular MCP-1-induced protein (MCPIP1) in Mφs. MCPIP1 mediates Mφs’ polarization and protection via dual catalytic activities. Additionally, we found that CCL2 induces MCPIP1 expression via the JAK2-STAT3 signaling pathway. Taken together, our results indicate that increased CCL2 expression in MM patients’ BM polarizes Mφs toward the M2-like phenotype and promotes the protective effect of Mφs through MCPIP1, providing novel insight into the mechanism of Mφs-mediated drug resistance in MM.

## Introduction

Multiple myeloma (MM) is an incurable hematologic malignancy characterized by the accumulation of monoclonal plasma cells in the bone marrow (BM)^1^. Despite the introduction of novel chemotherapy agents, chemoresistance remains the major problem in clinical management of MM^2^.

Regardless, the mechanism of MM chemoresistance has not yet been fully elucidated. Some studies have shown that MM cells possess clonal heterogeneity, and such mutations may result in resistance to chemotherapy^3^. In addition, other studies have reported that the BM plays an essential role in MM chemoresistance^4,5^, and that the interaction of MM cells with different cell components of the tumor microenvironment is important for tumor growth and chemoresistance^6^.

Macrophages (Mφs) are prominent components in the BM microenvironment of MM^7,8^. We previously found that Mφs protect MM cells from drug-induced apoptosis^7^. In addition, Mφs provide a favorable microenvironment for MM cells via crosstalk with other stromal cells and may also promote MM chemoresistance through Mφs-MM cells interaction^8^. Mφs possess great plasticity and can differentiate into different functional states according to microenvironmental signals^9^. Mφs can be classified into two major polarized states: M1-Mφs, which have remarkable tumoricidal activity; and M2-Mφs, which generally suppress antitumor immunity^10^. Many in vivo studies have indicated that tumor-associated Mφs (TAMs) are often educated to develop M2-like phenotypes in advanced stages of cancer^11,12^. Nonetheless, how Mφs are polarized toward the M2-like phenotype and whether the M2-like phenotype is associated with the protective effect of Mφs have not yet been fully defined.

Our previous study showed that the chemokine CCL2 promoted Mφs’ infiltration in the MM-BM microenvironment and encouraged Mφs’ proliferation^13^. CCL2, also known as MCP-1, is a member of the CC family of chemokines with an affinity for the receptor CCR2^14^. CCL2 has been shown to be a critical modulator of inflammation and recruitment of monocytes, NK cells, and T-cell subpopulations in numerous diseases^15,16^. In addition to regulating immune cell migration, CCL2 contributes to increased angiogenesis and bone resorption, two clinical features often observed in MM^17,18^. Some studies have shown that CCL2 plays a role in educating Mφs to become M2-like Mφs^19^, but many other studies have indicated that CCL2 is a marker of M1-like Mφs^20^.

In this study, we aimed to investigate the significance of CCL2 in Mφs-mediated MM chemoresistance. We performed mechanistic studies to further elucidate how CCL2 regulates Mφs’ function with regard to MM cells in vitro and in vivo. Our results provide new insight into the mechanism of drug resistance in MM.

## Materials and methods

### Cells

Human MM cell lines ARP-1, RPMI-8226, MM.1S, CAG, JJN3, and OPM2 were generously provided by Dr. Qing Yi (Center for Hematologic Malignancy, Research Institute, Houston Methodist, Houston, TX, USA) and cultured in RPMI-1640 medium containing 10% fetal bovine serum (FBS, Thermo Fisher Scientific, Gibco, Waltham, MA, USA) and 1% l-glutamine at 37 °C in 5% CO_2_ in air. Conditioned medium of MM cells was acquired from culture supernatants, which were seeded at 5 × 10^5^ cells/mL for 24 h.

PBMCs(peripheral blood mononuclear cells) were isolated from healthy donors after obtaining informed consent. Human Mφs were generated from PBMCs as previously described^7^. Briefly, monocytes were incubated in 6-well plates for 1–2 h at 37 °C; nonadherent cells were removed, and the adherent monocytes were incubated for 5–7 days in medium containing M-CSF (20 ng/ml; R&D Systems, MN, USA). Before use, the Mφs were phenotyped by morphological analysis, and the molecular marker CD14, CD68 were also detected (Supplementary Fig. 4A–C).

MM cells were cocultured with Mφs at a 1:1 ratio for 24 h with bortezomib (10 nM, Selleckchem, TX, USA) or melphalan (15 μM, MedChemExpress, NJ, USA). Then suspended MM cells were collected for functional assays to determine the protective effect of Mφs on bortezomib/melphalan-induced MM cell apoptosis. In some experiments, MM cells were cultured in Transwell inserts (0.4 μm pore size, Corning Inc., Tewksbury, MA, USA).

For inhibition experiments, Mφs were preincubated with Stattic (MedChemExpress, NJ, USA) for 2 h before rhCCL2 (50 ng/ml, R&D System, MN, USA) treatment. A neutralizing anti-CCL2 antibody (αCCL2, 50 μg/ml) was purchased from R&D Systems, MN, USA.

### Transient siRNA transfection

A scrambled nontargeting siRNA and one siRNA targeting MCPIP1 (MCPIP1-Homo-1694:5′GGUCUGAACCAUACCCACUTT-3′) were obtained from GenePharm, Shanghai, China. The siRNAs were transfected into cells according to the manufacturer’s protocol. Briefly, attached Mφs were incubated in Opti-MEM (catalog number 11058-021, Invitrogen, Carlsbad, CA, USA) with a complex of MCPIP1 siRNA or control siRNA and Lipofectamine 2000 transfection reagent (catalog number 11668027, Invitrogen, Carlsbad, CA, USA) for 24 h. The medium containing the siRNA transfection reagent complexes was then aspirated and replaced with RPMI-1640 medium and 10% FBS for 24 h before functional studies.

### Lentiviral transfection

For wild-type MCPIP1 and D141N mutant MCPIP1 overexpression, homo wild-type (WT) MCPIP1 cDNA or homo D141N mutant MCPIP1 cDNA and the GFP gene were inserted into the pCDH-CMV-EF1-T2A-Puro lentiviral vector. Lentiviral vectors were purified and then transfected into 293T cells, and lentiviral particles were collected after 48 h. Attached Mφs were transduced with LV-WT MCPIP1 or LV-D141N MCPIP1 (multiplicity of infection: 50) lentiviral vectors in the presence of polybrene (5–10 µg/mL) for 12 h. The empty vector (pCDH-CMV-EF1-T2A-Puro) was used as the negative control. MCPIP1 protein expression in the cells was examined by Western blotting after viral infection for 48 h.

### Quantitative real-time PCR

Total mRNA was isolated from cells using RNAiso^TM^ PLUS (TaKaRa, Shiga, Japan), and cDNA synthesis was performed using a PrimeScript^TM^ RT Reagent Kit with DNA Eraser (TaKaRa, Shiga, Japan). Quantitative real-time polymerase chain reaction (qRT-PCR) was carried out with SYBR Premix Ex Taq II (TiRNaseH Plus) (Takara, Shiga, Japan) and a Bio-Rad CFX96 real-time system (Bio-Rad, Hercules, CA, USA) according to the manufacturer’s instructions. Data were analyzed using the relative standard curve method and normalized to GAPDH. The primer sets used for these analyses are summarized in Supplementary Table 1.

### Western blot analysis

Cells were collected and extracted with lysis buffer containing a protease and phosphatase inhibitor cocktail (Thermo Fisher Scientific, Waltham, MA, USA). Equal amounts of protein were separated by sodium dodecyl sulfate polyacrylamide gel electrophoresis and then transferred onto polyvinylidene difluoride membranes (Merck Millipore, Darmstadt, Germany). The membranes were blocked with 5% nonfat milk for 1–2 h and then incubated with corresponding primary antibodies overnight at 4 °C. The membranes were washed with Tris-buffered saline with Tween 20 (TBST) and incubated with an horseradish peroxidase (HRP)-conjugated anti-rabbit or anti-mouse antibody in TBST for 1 h at room temperature. The membranes were washed three times with TBST, and the protein bands were detected using a ChemiDoc^TM^ MP Imaging System (Bio-Rad) and an enhanced chemiluminescence detection kit (Biological Industries, Israel, Beit Haemek Ltd., Kibbutz Beit Hamek, Israel). Primary antibodies, including anti-p-AMPK, -caspase-3, -caspase-9, -Bad, -Bcl2, -Bax, -Bcl-xl, -STAT3, -p-STAT3 (Tyr705), -pSTAT3 (Ser727), -JAK2, -p-JAK2 (Y1008), and -SOCS3 were obtained from Cell Signaling Technology (MA, USA). Anti-iNOS and -PARP-1 antibodies were purchased from Abcam (Cambridge, UK). An anti-GAPDH antibody was purchased from Sigma-Aldrich, Billerica (MA, USA), and an anti-MCPIP1 antibody was purchased from Santa Cruz Biotechnology, CA, USA.

### Proteome profiler human phospho-kinase antibody array

Proteome profiler human phospho-kinase antibody array was utilized according to the manufacturer’s protocol. In this array, 46 captured antibodies or control antibodies against human phosphorylated kinases are spotted in duplicate on nitrocellulose membranes.

Before harvesting, Mφs were treated with PBS or rhCCL2 for 24 h and lysed using lysis buffer (ARY003, Proteome Profiler^TM^, R&D Systems, Minneapolis, MN, USA). The total protein concentration of each sample was quantified using a Bio-Rad DC protein assay kit II (Cat #500-0112, Bio-Rad Laboratories, Philadelphia), and 150 µg/mL was used for the array. The lysates were incubated overnight with the array membranes, and after 24 h, they were washed to remove any unbound proteins. Further incubation was performed with a cocktail of biotinylated detection antibodies for 2 h at room temperature. The membranes were then exposed to streptavidin–HRP for 30 min. After a final wash, the proteins bound to the membrane were detected by exposure to an enhanced chemiluminescent reagent for 1 min. Chemiluminescent images were captured using a Syngene G-BOX (G: BOX-CHEMI_XL1.4, Syngene, Cambridge, UK). To quantify activation levels of the proteins, the integrated optical density (IOD) of each spot was assessed at increasing exposure using a Bio-Rad ChemiDoc station; the IOD values were corrected for background signals. To compare different membranes, the values were normalized to those of the positive controls on each membrane, and the protein expression levels were then quantified. The experiment was performed twice to confirm the results obtained.

### Flow cytometry

Apoptosis was detected by staining cells with Annexin V-FITC/propidium iodide (Dojindo, Kumamoto, Japan) according to the manufacturer’s instructions. CD14Ab (Biolegend, CA, USA) staining was used to exclude Mφs from the analysis.

Expression of active caspase-3 was measured using a Fluorescein Active Caspase-3 Staining Kit (Invitrogen, Carlsbad, CA, USA). Expression of CD206, CD163, and CD86 was measured by direct immunofluorescence using PE-conjugated CD206 (Biolegend, CA, USA) and CD163 (Biolegend, CA, USA) and APC-conjugated CD86 (Biolegend, CA, USA). An isotype control was used to exclude nonspecific signals.

For intracellular CCL2 staining, cells were stimulated with Leukocyte Activation Cocktail (BD Bioscience, CA, USA) for 4 h at 37 °C. The cells were then fixed and permeabilized with Fixation Buffer and Intracellular Staining Perm Wash Buffer (Biolegend, CA, USA) according to the manufacturer’s instructions. Next, the cells were stained with a PE-mouse anti-human MCP-1 antibody (BD Pharmingen, San Diego, CA, USA). Data were acquired with a FACScan flow cytometer (BD Biosciences, San Diego, CA, USA) and analyzed using FlowJo 7.6.1.

### Cell proliferation assay

A CCK-8 proliferation assay (Dojindo, Kumamoto, Japan) was used to assess MM cell proliferation. A total of 1 × 10^4^ MM cells/well were seeded in 96-well plates and cultured for the indicated times. The cells were treated with CCK-8 solution (10 µL) for another 2 h at 37 °C, and absorbance was measured at 450 nm using a microplate reader (Bio-Rad, Model 680).

### Enzyme-linked immunosorbent assay

CCL2 in peripheral serum and cell culture supernatants (kits from Biolegend, CA, USA) and IL-10 and TNF-α secreted by Mφs (kits from Biolegend, CA, USA) were analyzed by ELISA according to the suppliers’ instructions.

### RNA sequencing

RNA sequencing was performed following Illumina mRNA Sequencing Sample Preparation Guide (Ilumina, CA, USA). Mφs generated from PBMCs of 2 independent healthy donors (Mφs-1, Mφs -2) were treated with rhCCL2 (50 ng/mL) for 24 h. Total RNA was then extracted using Redzol reagent (SBS Genetech Inc., Beijing, China) and quantified (NanoDrop spectrophotometer, Thermo Fisher Scientific), with quality control using the Agilent BioAnalyzer 2100 system (Agilent Technologies Inc., Beijing, China). Sequencing cluster generation and sequencing were performed with Genome Analyzer IIx (Illumina) at Ruibo Biotechnology Company (Guangzhou, China).

### Immunohistochemistry and immunofluorescence

Formalin-fixed, paraffin-embedded BM biopsy samples from MM patients were examined by immunohistochemistry to detect expression of CCL2. All 12 cases were histopathologically and clinically diagnosed as new untreated MM, and the patients received four courses of PCD (bortezomib + cyclophosphamide + dexamethasome) therapy at The First Affiliated Hospital, School of Medicine, Zhejiang University from January to May 2017. All patients consented to participate in the study, and the study was approved by the Research Ethics Committee of the First Affiliated Hospital, School of Medicine, Zhejiang University. The degree of immunostaining was evaluated using H-Score. Anti-CD138, anti-cleaved caspase-3, and anti-MCPIP1 antibody immunofluorescence analysis was performed. Image-pro plus 6.0 software (Media Cybernetics, Inc., Rockville, MD, USA) was used for quantification of cleaved caspase-3 signals. The quantitative value of areal density = IOD (integral optical density)/area (pixels area), presenting positive expression of cleaved caspase-3 in the captured image. Antibodies against cleaved caspase-3 and CD138 were obtained from Proteintech Group Inc., IL, USA. An anti-MCPIP1 antibody was purchased from Santa Cruz Biotechnology and an anti-CCL2 antibody from Abcam (Cambridge, UK).

### MM xenograft model

Four-week-old female NSG mice were obtained from the Model Animal Research Center of Nanjing University (Nanjing, China) and housed in the animal facility of Zhejiang University School of Medicine. The Tab of Animal Experimental Ethical Inspection of the First Affiliated Hospital, School of Medicine, Zhejiang University approved the procedures and protocols of all experiments. ARP-1 (5 × 10^6^) cells were injected subcutaneously into the right flanks of the mice. After 7 days, when palpable tumors (>5 mm) had developed, some of the mice were injected intraperitoneally with bortezomib (2 µg/mouse, every 3 days) for 1 week or with PBS as a control. For some mice, differently treated Mφs were injected into the tumor mass, and some mice received CCX140-B (MedChemExpress, NJ, USA) by oral gavage (500 µg/mouse/day) every day for 1 week. Tumor size was monitored daily with calipers, and upon sacrifice, CD14+ cells were purified from the tumors using anti-CD14-coated magnetic microbeads (Miltenyi Biotec, Bergisch Glabach, Germany). These cells were used for RNA extraction.

### Statistical analysis

GraphPad Prism 6 and Excel were used for all statistical analyses. Values represent the means ± SD for at least three independent experiments performed in triplicate. Significant differences between experimental groups were determined using a two-tailed Student’s *t* test and one-way analysis of variance where appropriate. All *P* values < 0.05 were considered statistically significant. The significance of *P* values is *P < 0.05; **P < 0.01, ***P < 0.001, NS : not significant.

## Results

### Clinical significance of CCL2 expression in MM

**Fig. 1:**
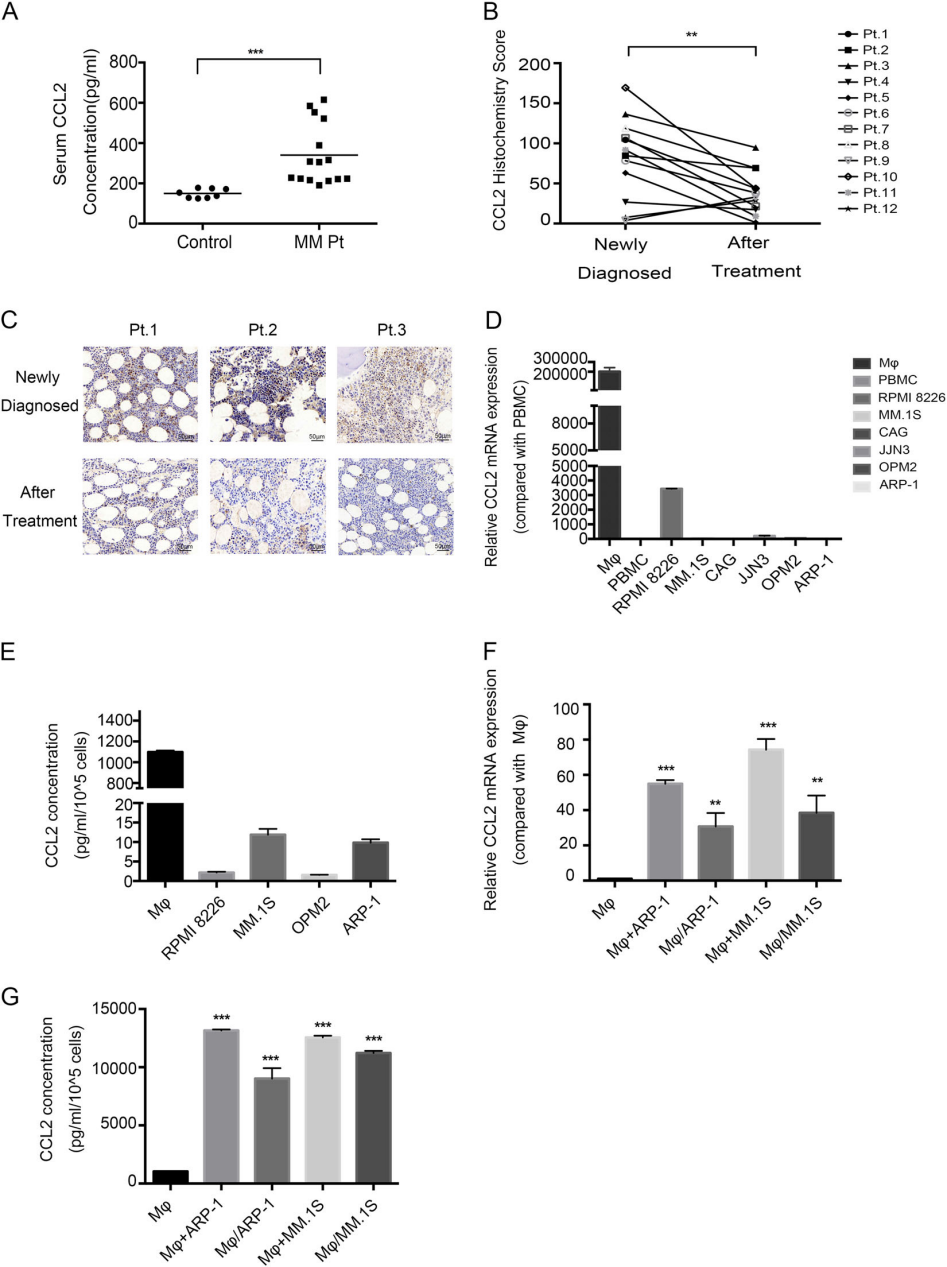
Clinical significance and expression of CCL2 in MM. **a** Levels of CCL2, as measured by ELISA, in the peripheral serum of healthy donors (control) and MM patients (MM Pt). Samples from 8 and 15 healthy donors and MM patients, respectively, were used. **b** The histochemistry score for CCL2 was detected using BM biopsy samples when the patients were newly diagnosed or after treatment (median H-score from 88.104320 to 29.8143). Pt.1 to Pt.12 represent 12 independent patients. **c** Immunohistochemical analysis of CCL2 expression in 3 representative BM biopsies (Pt.1 to Pt.3). The top panel shows CCL2 expression when patients were newly diagnosed, and the bottom panel shows CCL2 expression after the same patient received therapy. Scale bars, 50 μm. **d** Ratio of mRNA expression of CCL2 in MM cell lines (RPMI 8226, MM.1 S, CAG, JJN3, OPM2, ARP-1), PBMCs and macrophages (Mφs) by RT-PCR. **e** A total of 1 × 10^5^ cells were cultured in 1 mL of medium for 24 h, followed by ELISA analysis of CCL2 expression in cell culture supernatants. **f, g** Mφs were cultured alone (Mφs) or cocultured with MM cells directly (Mφs + ARP-1, Mφs + MM.1S) or through Transwell chambers (Mφs/ARP-1, Mφs/MM.1S) for 24 h. Then, the fresh median was changed, and the Mφs were cultured for another 24 h, followed by RT-PCR **f** and ELISA **g** to detect CCL2 expression. Summarized data from at least three independent experiments are shown. Values are presented as means ± SD. *P < 0.05; **P < 0.01; ***P < 0.001

We first examined CCL2 expression in peripheral serum from newly diagnosed MM patients and healthy donors using ELISA. As shown in Fig. 1a, CCL2 could be detected in samples from all patients and donors, with a much higher concentration in the former. Interestingly, we found that after the newly diagnosed patients received four courses of PCD combined therapy, CCL2 expression in their BM was significantly decreased (Fig. 1b, c). Based on the clinicopathologic characteristics we collected, almost all of these patients were in remission as a result of PCD combined therapy (Table 1). These results strongly suggest that CCL2 expression may be tightly associated with the treatment status of MM patients.

**Table 1:** Patient characteristics

Variables	Newly diagnosed (*n* = 12)	After PCD treatment (*n* = 12)	*P*
Median age, years (range)	59 (39–74)		
Sex (M/F)	4/8		
*Durie-Salmon staging, n (%)*			
Stage IA	–	3 (25%)	
Stage IIA	1 (8.33%)	–	
Stage IIB	–	–	
Stage IIIA	9 (75%)	8 (66.67%)	
Stage IIIB	2 (16.67%)	1 (8.33%)	
*ISS, n (%)*			
Stage I	–	–	
Stage II	7 (58.33%)	12 (100%)	
Stage III	5 (41.67%)	–	
*Median glb (g/L), (range)*	42.65 (17.4–89.8)	22.05 (15.7–42.4)	0.015
≥35 g/L	6 (50%)	1 (8.33%)	
<35 g/L	6 (50%)	11 (91.67%)	
*Median β2-microglobulin (mg/L), (range)*	5.14 (2.66–22.4)	2.355 (1.74–3.8)	0.012
≥5.5 mg/L	6 (50%)	–	
<5.5 mg/L	6 (50%)	12 (100%)	
*Median Hb (g/L), (range)*	107 (67–140)	124 (100–137)	0.059
≥105 g/L	7 (58.33%)	11 (91.67%)	
<105 g/L	5 (41.67%)	1 (8.33%)	
*Median Cre (μmol/L), (range)*	61.5 (55–400)	58.5 (36–166)	0.199
≥104 μmol/L	3 (25%)	1 (8.33%)	
<104 μmol/L	9 (75%)	11 (91.67%)	
*Median Ca* ^*++*^ *(mmol/L) (range)*	2.305 (1.95–4.19)	2.145 (1.87–2.28)	0.087
≥2.54 mmol/L	3 (25%)	–	
<2.54 mmol/L	9 (75%)	12 (100%)	
*LDH(U/L) (range)*	170 (145–300)	202 (173–266)	0.342
Normal	10 (83.33)	9 (75%)	
High	2 (16.67%)	3 (25%)	
*M protein subtype, n (%)*			
IgG kappa	1 (8.33)		
IgG lamda	2 (16.67%)		
IgA kappa	3 (25%)		
IgA lamda	2 (16.67%)		
Kappa light chain	2 (16.67%)		
Lamda light chain	2 (16.67%)		
Median plasma cells in bone marrow (%), (range)	20 (0.5–58)	2 (0–11)	0.002

Characteristics of the patients included in the study. PCD: Bortezomib + Cyclophosphamide + Dexamethasone

### MM cells induced CCL2 expression in Mφs

We evaluated CCL2 expression in human MM cell lines, Mφs and PBMCs. qRT-PCR, ELISA, and flow cytometry results all revealed that MM cell lines and PBMCs barely expressed CCL2 but that Mφs highly expressed CCL2 (Fig. 1 d, e and Supplementary Fig. 1A). We also identified the expression of CCL2 in CD68+ Mφs and CD138+ MM cells from MM patients’ BM biopsies using immunofluorescence (Supplementary Fig. 1b), showing CCL2 was mainly expressed by Mφs. To determine whether MM cells affect CCL2 expression in Mφs, we cocultured Mφs with MM cells directly or through Transwell chambers for 24 h. According to qRT-PCR, ELISA, and flow cytometry, MM cells significantly induced CCL2 expression in Mφs (Fig. 1f, g and Supplementary Fig. 1C).

### CCL2 did not affect the proliferation and drug responses of MM cells

To determine the effect of increased CCL2 levels in the BM microenvironment in MM, we conducted CCK-8 assays to examine whether CCL2 promotes MM cells proliferation. As shown in Supplementary Fig. 1D, there was no difference between groups treated or not with rhCCL2. We also performed flow cytometry to evaluate whether CCL2 affects the response of MM cells to bortezomib. Based on the results, bortezomib induced similar levels of apoptosis in MM cells (Supplementary Fig. 1E), suggesting that rhCCL2 does not affect the drug response of MM cells. To determine why rhCCL2 had little effect on MM cells, we measured CCR2 expression in human MM cell lines by flow cytometry and found scarce expression of CCR2 (Supplementary Fig. 1F). This finding indicates that increased levels of CCL2 in MM BM may play an important role via other cell components.

### rhCCL2-treated Mφs are more effective at protecting MM cells from melphalan- and bortezomib-induced apoptosis

**Fig. 2:**
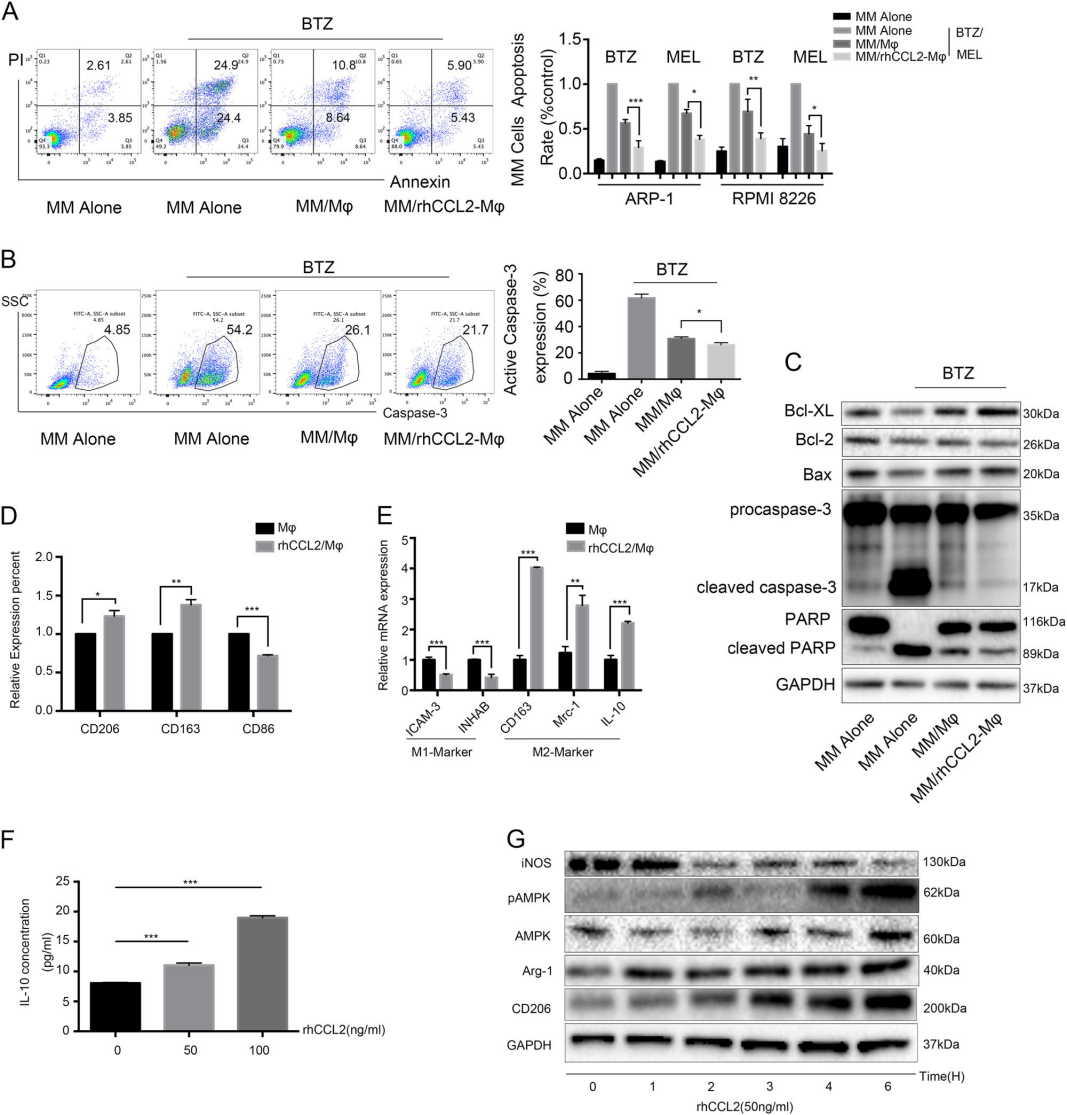
CCL2 promotes Mφs to mediate MM multidrug resistance and polarizes Mφs toward the M2 phenotype. **a** Percentage of bortezomib (BTZ, 10 nM) or melphalan (MEL, 15 μM)-induced apoptotic MM cells under different culture conditions: cultured alone (MM Alone), cocultured with Mφs (MM/Mφs), and cocultured with rhCCL2 pretreated Mφs (MM/rhCCL2-Mφs). A representative flow cytometry analysis showed the apoptosis of ARP-1 cells induced by BTZ and summarized results from at least three independent experiments are shown. Values are presented as means ± SD. **b** A representative flow cytometry analysis showed the percentage of MM cells with active caspase-3 induced by bortezomib (BTZ, 10 nM). The summarized results are from at least three independent experiments. Values are presented as means ± SD. **c** Western blotting analysis showed the bortezomib (BTZ, 10 nM)-induced cleavage and activation of PARP and caspase-3 in ARP-1 cells under different culture conditions. **d** Mφs were exposed to rhCCL2 for 24 h (rhCCL2/Mφs), followed by flow cytometry to detect the percentages of surface markers of CD206, CD163 and CD86 of Mφs and **e** RT-PCR was used to determine the ratio of mRNA expression of the indicated M1 or M2 signature genes in Mφs and **f** ELISA to examine the concentration of IL-10 in culture supernatants. The summarized results are from at least three independent experiments. Values are presented as means ± SD. **g** Immunoblot analysis of iNOS, phosphor-AMPK (pAMPK), AMPK, Arg-1, and CD206 in Mφs treated with rhCCL2 for the indicated times. Student’s *t* test **a**, **d**–**f**, one-way ANOVA **b**, *P < 0.05; **P < 0.01, ***P < 0.001

Our previous studies showed that BM-infiltrated Mφs can induce drug resistance in MM^7^. To determine whether CCL2 promotes this protective effect of Mφs, we treated Mφs with rhCCL2 for 24 h to generate rhCCL2-Mφs. As shown in Fig. 2a, coculture of MM cells with Mφs protected MM cells from bortezomib- and melphalan-induced apoptosis, and rhCCL2-Mφs were more effective than Mφs. Flow cytometry also revealed that when MM cells were cocultured with rhCCL2-Mφs, bortezomib induced fewer MM cells to express active caspase-3 than when cocultured with Mφs (Fig. 2b). Additionally, Western blotting showed that bortezomib treatment resulted in cleaved caspase-3 and cleaved PARP in MM cells. However, when ARP-1 cells were cocultured with Mφs, bortezomib-induced PARP and caspase-3 cleavage were highly repressed, and rhCCL2-Mφs were more effective at this repression (Fig. 2c). Taken together, these results demonstrate that rhCCL2 promotes Mφs to protect myeloma cells from melphalan-and bortezomib-induced apoptosis by inhibiting caspase activation.

### rhCCL2 polarized Mφs toward the M2-like phenotype in vitro

According to previous studies, Mφs in different states of polarization exert different functions on MM cells’ survival and tumor growth^19^. To test our hypothesis that the polarization of Mφs is associated with their capacity to protect MM cells, we generated IL4-Mφs and LPS + IFNγ-Mφs and evaluated their protective capacity. IL4-Mφs expressed higher levels of the M2 surface marker CD206 than Mφs and LPS + IFNγ-Mφs, indicating that these cells were in different polarization states (Supplementary Fig. 2A). Compared with cells cocultured with Mφs or LPS + IFNγ-Mφs, fewer MM cells cocultured with IL4-Mφs underwent apoptosis induced by bortezomib (Supplementary Fig. 2B). This result demonstrates that M2-like Mφs are more effective at protecting MM cells from bortezomib-induced apoptosis.

We next sought to determine whether rhCCL2 is able to polarize Mφs toward the M2-like phenotype. Flow cytometry analysis of Mφs surface markers showed that rhCCL2 upregulated expression of CD206 and CD163 (classic markers of M2-like Mφs) and downregulated that of CD86 (classic surface marker of M1-like Mφs) (Fig. 2d). Moreover, based on qRT-PCR, rhCCL2-Mφs expressed lower mRNA levels of M1-like genes (ICAM-3 and INHAB) and higher levels of M2-like genes (CD163, Mrc-1, and IL-10) (Fig. 2e). ELISA was then conducted to detect secretion of IL-10, a classic anti-inflammation factor secreted by M2-like Mφs. As shown in Fig. 2f, rhCCL2-Mφs secreted more IL-10 than did untreated Mφs. In addition to changes in receptor surface expression and cytokine secretion, Mφs polarization was associated with a shift in energy metabolism, and adenosine 5′-monophosphate-activated protein kinase (AMPK) was central in this regulation^19,21,22^. These studies showed that M2-like Mφs were related to rapid AMPK phosphorylation and AMPK activation could drive IL-10 production in Mφs. We next performed Western blotting and found that rhCCL2 time-dependently activated AMPK by increasing T172 phosphorylation levels. Expression of Arg-1 and CD206 was also upregulated along with the exposure of rhCCL2. All these results suggest that rhCCL2 effectively polarizes Mφs toward the M2-like phenotype, with a stronger ability to protect MM cells from bortezomib-and melphalan-induced apoptosis.

### A CCR2 inhibitor suppressed the protective effect of Mφs in vivo

**Fig. 3:**
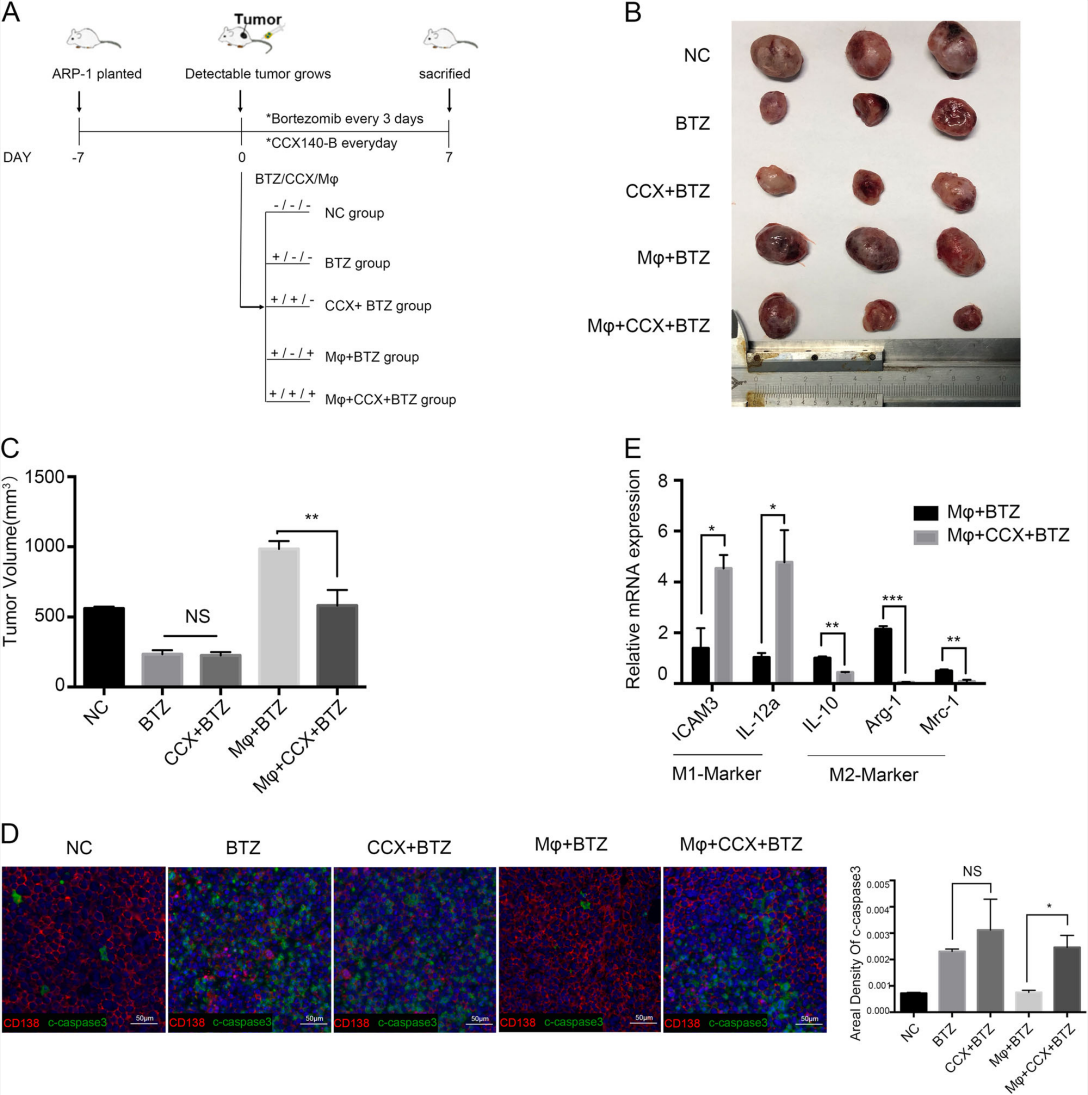
A CCR2 inhibitor disrupts the protective effect of Mφs in vivo. **a** The workflow of the experiment in vivo. NSG mice were subcutaneously inoculated in the flank with 5 × 10^6^ ARP-1 cells. When these mice bearing detectable tumors (Day 0), they were assigned randomly to five groups (*n* = 5 per group), with 0.4 × 10^5^ Mφs injected into the tumor mass for two groups (Mφs + BTZ group, Mφs + CCX + BTZ group). Bortezomib (BTZ) was administered via intraperitoneal injection at a dose of 2 µg/mouse every 3 days. CCX140-B (CCX) was administered via oral gavage (500 µg/mouse) every day for 1 week, and tumor growth was monitored. **b**, **c** Graph showing the tumor volume at the end point of the trial. **d** Immunofluorescence analysis of CD138 (Red) and cleaved caspase-3 (Green) expression in tumor tissues from mice of different groups. Scale bar, 50 μm. Areal density of cleaved caspase-3 along intratumoral areas is represented on the right. Data show the mean ± SD of at least three mice per group. **e** Ratio of mRNA expression of the indicated M1 or M2 signature genes in CD14 + Mφs isolated from the tumor masses determined by RT-PCR. The data show means ± SD. NS not significant. *P < 0.05; **P < 0.01, ***P < 0.001

Next, we performed in vivo experiments to determine the role of CCL2 in the MM microenvironment. Figure 3a shows the workflow of the experiment. CCX140-B is a specific CCR2 inhibitor, and as illustrated in Fig. 3b, c, there were no significant difference in the tumor volumes between the BTZ and CCX + BTZ groups, which indicated that blocking the CCL2–CCR2 axis in MM cells scarcely influenced the effect of bortezomib. However, in the presence of Mφs, CCX140-B significantly hindered the growth of tumors, suggesting that blocking the CCL2–CCR2 axis in Mφs disrupted the protective effect of Mφs in vivo. Immunofluorescence results also revealed that the tumors in the Mφ + CCX + BTZ group had lower CD138 levels and higher active caspase-3 levels than in the Mφ + BTZ group; thus, with CCX140-B treatment, more MM cells underwent apoptosis upon bortezomib treatment (Fig. 3d). We then explored the effect of CCR2 blockade on Mφs polarization by separating CD14+ cells from the tumor mass and using quantitative RT-PCR to detect expression of Mφs polarization-associated genes. The results showed that Mφs from Mφ + CCX + BTZ group mice expressed significantly lower levels of IL-10, Arg-1, and Mrc-1, and higher levels of ICAM-3 and IL-12α than did Mφs from Mφ + BTZ group mice (Fig. 3e). Taken together, our results indicate that CCL2 is associated with the protective effect of Mφs and may alter the polarization status of these cells.

### CCL2 induced MCPIP1 expression in Mφs

**Fig. 4:**
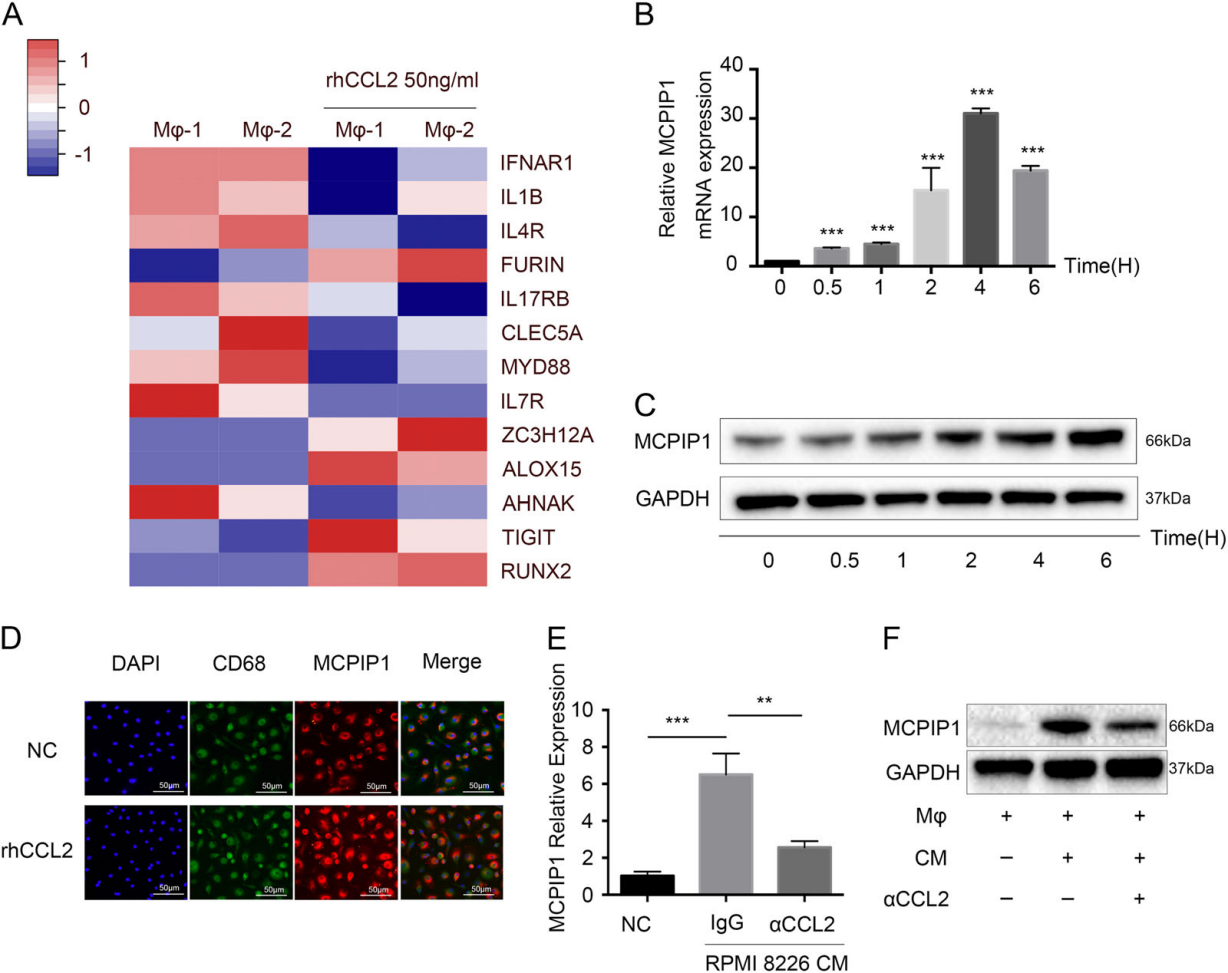
CCL2 induces expression of MCPIP1 in Mφs. **a** Mφs generated from PBMCs of two independent healthy donors (Mφs-1 and Mφs-2) were treated with rhCCL2 (50 ng/ml) for 24 h. RNA was extracted for RNA sequencing. Heat maps illustrate the log_2_-fold change of some immune-related genes. **b** Mφs were exposed to rhCCL2 (50 ng/mL) for the indicated times, followed by RT-PCR and Western blotting **c** to determine expression of MCPIP1. **d** Mφs were treated with rhCCL2 (50 ng/mL) for 24 h. Immunofluorescence was performed to detect expression of MCPIP1 in Mφs. Scale bar, 50 μm. **e**, **f** Mφs were treated with RPMI-8226 CM for 24 h with or without αCCL2. qRT-PCR **e** and Western blotting **f** were performed to detect MCPIP1 expression. Data are presented as means ± SD of at least three independent experiments. *P < 0.05; **P < 0.01, ***P < 0.001

To explore the mechanism by which rhCCL2-Mφs are more effective at protecting MM cells, we cultured Mφs with rhCCL2 for 24 h and detected the global transcriptional profile of these cells by RNA sequencing (RNA-Seq). The change in some immune-related genes between Mφs and rhCCL2-Mφs is depicted in Fig. 4a. Among these genes, we were interested in the change of ZC3H12A, which is also called MCPIP1, has been uncovered to act as a negative regulator of inflammation^23^. MCPIP1 was initially identified when gene expression changes in human PBMCs treated with CCL2 were analyzed by a genomic approach with gene arrays^24^, and this study named the most highly induced expressed sequence tag (EST) MCP-1-induced protein (MCPIP1). Considering the close connection between CCL2 and MCPIP1, we attempted to determine whether MCPIP1 is involved in the enhanced protective effect of rhCCL2-Mφs or whether it promotes M2 polarization.

As shown in Fig. 4b–d, rhCCL2 induced expression of MCPIP1 in Mφs. We also found that the culture medium of MM cells (RPMI 8226) significantly upregulated MCPIP1 expression in Mφs. In the presence of αCCL2, a neutralizing antibody, MCPIP1 expression was downregulated, indicating that increased CCL2 expression induced by MM cells also effectively triggered MCPIP1 expression in Mφs (Fig. 4e, f).

### The enhanced protective effect of rhCCL2-Mφs relied on induced expression of MCPIP1

**Fig. 5:**
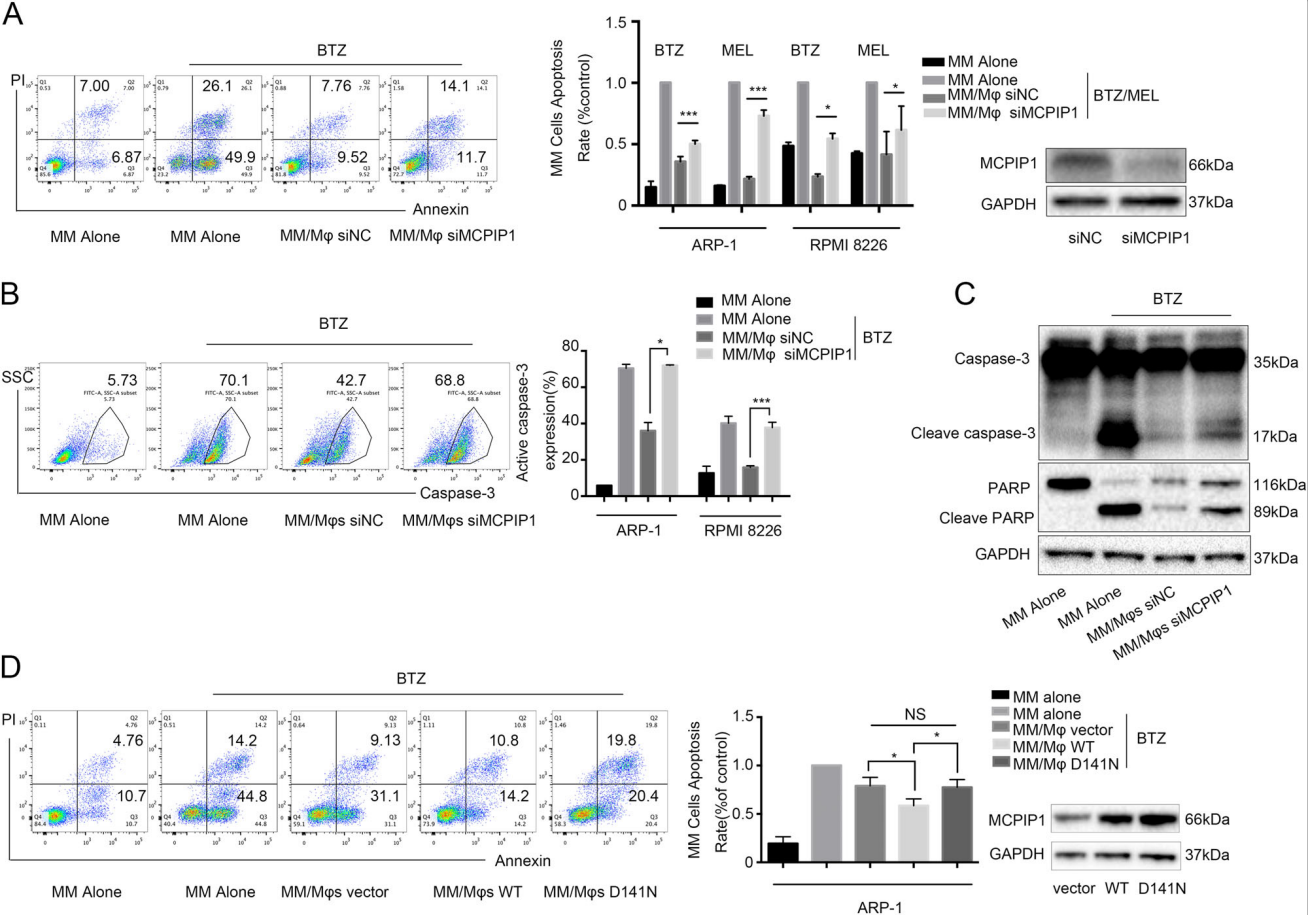
MCPIP1 is crucial for Mφs-mediated protection of MM cells via dual catalytic activities. **a** Percentage of bortezomib (BTZ, 10 nM) or melphalan (MEL, 15 μM)-induced apoptotic MM cells under different culture conditions: cultured alone (MM Alone), cocultured with Mφs transfected with a scrambled nontargeting siRNA (MM/Mφs siNC), and cocultured with Mφs transfected with MCPIP1-specific siRNA (MM/Mφs siMCPIP1). A representative flow cytometry analysis showed the apoptosis of ARP-1 cells induced by BTZ, and summarized results from at least three independent experiments are shown. Values are presented as means ± SD. A representative western blotting analysis showed the knockdown efficiency of the MCPIP1-specific siRNA in Mφs. **b** A representative flow cytometry analysis showed the percentage of MM cells with active caspase-3 induced by bortezomib (BTZ, 10 nM). The summarized results are from at least three independent experiments. Values are presented as means ± SD. **c** Western blotting analysis showed the bortezomib (BTZ, 10 nM)-induced cleavage and activation of PARP and caspase-3 in ARP-1 cells under different culture conditions. **d** Percentage of bortezomib (BTZ, 10 nM)-induced apoptotic ARP-1 cells in different culture conditions: cultured alone (MM Alone), cocultured with Mφs transfected with the empty lentiviral vector (MM/Mφs vector), cocultured with Mφs transfected with wild-type MCPIP1 lentivirus (MM/Mφs WT), and cocultured with Mφs transfected with the D141N-MCPIP1 lentivirus (MM/Mφs D141N). Representative and summarized results showing the apoptosis rate of ARP-1 cells. A representative Western blotting analysis shows the MCPIP1 overexpression efficiency of the lentivirus in Mφs. The summarized results are from at least three independent experiments. Values are presented as means ± SD. NS: not significant. *P < 0.05; **P < 0.01, ***P < 0.001

To determine whether upregulated MCPIP1 expression promotes the observed protective effect, Mφs were transfected with MCPIP1-specific siRNA or a nonspecific scrambled control. As shown in Fig. 5a, when MM cells were cocultured with siMCPIP1-Mφs, the apoptosis rates induced by bortezomib or melphalan were much higher than those of cells cocultured with siNC-Mφs. This finding indicated that expression of MCPIP1 in Mφs affected their capacity to protect MM cells from melphalan- and bortezomib-induced apoptosis. Next, we conducted the same experiment using primary MM cells from a patient and found that siMCPIP1-Mφs possessed a poor capacity to protect primary MM cells upon bortezomib treatment (Supplementary Fig. 3A). In addition, we performed flow cytometry to assess the percentage of MM cells with activated caspase-3 induced by bortezomib (Fig. 5b), and the results indicated that knocking down MCPIP1 hindered the Mφs-mediated protection of MM cells from bortezomib-induced apoptosis. Moreover, Western blotting revealed that compared with siNC-Mφs, siMCPIP1-Mφs were inferior in suppressing bortezomib-induced activation and cleavage of caspase-3 and PARP in MM cells (Fig. 5c).

To rule out the possibility that transfection with the MCPIP1-specific siRNA affected Mφs survival, we conducted flow cytometry and Western blotting to assess apoptosis in siMCPIP1-Mφs and siNC-Mφs. As shown in Supplementary Fig. 3B, C, there were no differences in apoptosis between these cells, indicating that transfection with the MCPIP1-specific siRNA barely affected Mφs survival.

### MCPIP1 promoted the protective effect of Mφs via dual catalytic activities

MCPIP1 is known to have deubiquitinase and RNase activities, including anti-Dicer activity^25–27^. To determine whether these activities of MCPIP1 are involved in the enhanced protective effect of Mφs, we used lentiviruses to generate deletion mutants of MCPIP1. It has been shown that the D141N mutant of MCPIP1 inactivates both its RNase and DUB activities^28^. As shown in Fig. 5d, Mφs transfected with wild-type MCPIP1 exhibited better protective effects than did Mφs transfected with the lentivirus vector. Interestingly, when ARP-1 cells were cocultured with D141N-mutant MCPIP1 Mφs, more MM cells underwent apoptosis than when these cells were cocultured with wild-type MCPIP1 Mφs, and the apoptosis rates were as high as those when the cells were cocultured with Mφs transfected with the lentivirus vector. These results indicate that MCPIP1 overexpression promotes the protective effect of Mφs via its dual catalytic activities.

### The protective effect of Mφs relied on MCPIP1 expression in vivo

**Fig. 6:**
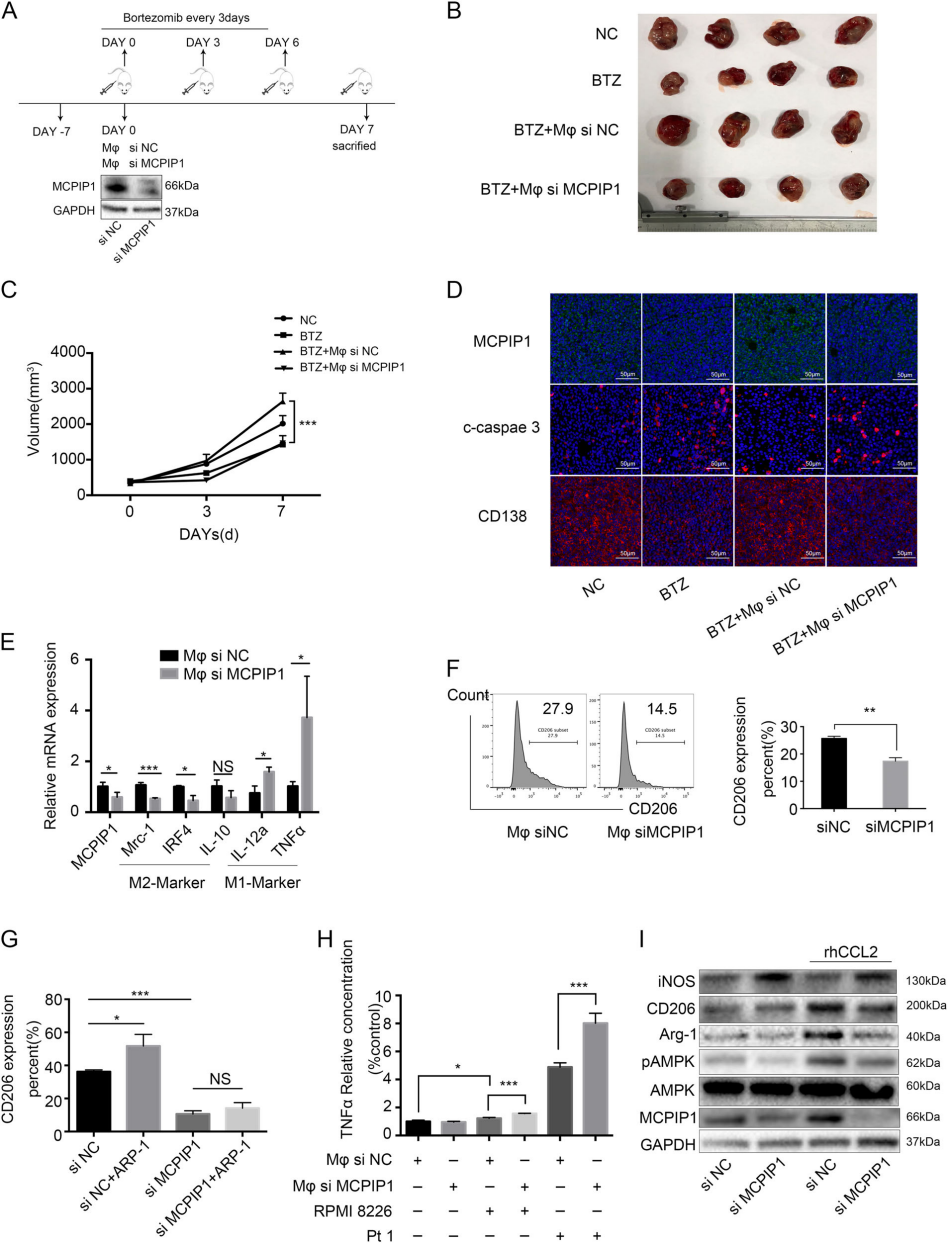
The protective effect of Mφs relies on MCPIP1 in vivo, and MCPIP1 is crucial for Mφs polarization. **a** The workflow of the experiment in vivo. NSG mice were subcutaneously inoculated in the flank with 5 × 10^6^ ARP-1 cells. When tumors were detectable (Day 0), the mice were assigned randomly to 4 groups (*n* = 6 per group), with Mφs that were differentially transfected (0.4 × 10^5^) injected into the tumor mass for two groups (Mφs siNC, Mφs siMCPIP1). A representative Western blotting analysis shows the knockdown efficiency of MCPIP1-specific siRNA in Mφs. Bortezomib (BTZ) was administered via intraperitoneal injection at a dose of 2 µg/mouse every 3 days, and tumor growth was monitored. **b** A representative image of tumor volumes at the end point of the trial **c** Mean tumor volumes over the time course. **d** Immunofluorescence analysis of MCPIP1, CD138, and cleaved caspase-3 expression in tumor tissues from mice of different groups. Scale bar, 50 μm. **e** Ratio of mRNA expression of the indicated M1 or M2 signature genes in murine Mφs isolated from the tumor masses by RT-PCR. **f** Representative and summarized results show CD206 expression measured by flow cytometry in murine Mφs isolated from the tumor mass. Values are presented as means ± SD. **g** Differently transfected Mφs (siNC, siMCPIP1) were cocultured with or without ARP-1 cells for 24 h, followed by flow cytometry to detect CD206 expression. The summarized results are from at least three independent experiments. Values are presented as means ± SD. **h** Differently transfected Mφs (siNC, siMCPIP1) were cocultured with or without RMPI 8226 cells and primary MM cells from a patient (Pt.1) for 24 h. The fresh medium was changed, and Mφs were cultured for another 24 h, followed by ELISA analysis to detect TNF-α expression. The summarized results are from at least three independent experiments. Values are presented as means ± SD. **i** Differently transfected Mφs (siNC, siMCPIP1) were exposed to rhCCL2 (50 ng/mL) for 24 h, and immunoblot analysis was conducted to determine expression of iNOS, CD206, Arg-1, pAMPK, AMPK, and MCPIP1. The data show means ± SD. NS not significant. *P < 0.05; **P < 0.01, ***P < 0.001

We next used an MM cell xenograft model to determine whether MCPIP1 plays an important role in promoting Mφs to protect MM cells in vivo. Figure 6a shows the workflow of the experiment. ARP-1 cells were first injected subcutaneously into NSG mice, and when the average tumor volume reached approximately 5 mm^3^, we injected either siNC-Mφs or siMCPIP1-Mφs into the tumor mass. In addition, the mice were treated with bortezomib every three days. As shown in Fig. 6b, c, tumor size measurements revealed that the siNC-Mφ group developed larger tumors than the siMCPIP1-Mφ group, suggesting that MCPIP1 has an important role in the protective effect of Mφs. Furthermore, tissue analysis showed that MM + siMCPIP1-Mφ tumors displayed low CD138 levels and high active caspase-3 levels. Consequently, mice in this group had a lighter tumor burden, and more myeloma cells underwent apoptosis upon bortezomib treatment (Fig. 6d). These data demonstrate that the protective effect of Mφs in vivo relies on MCPIP1.

### MCPIP1 was crucial for Mφs polarization

To compare the polarization state of siNC-Mφs and siMCPIP1-Mφs in vivo, we isolated leukocytes from the NSG mouse tumor masses and then conducted quantitative RT-PCR to determine the polarization state of Mφs. As shown in Fig. 6e, siMCPIP1-Mφs expressed greater mRNA levels of M1 Mφs-associated IL-12α and TNF-α and lower levels of M2 Mφs-associated Mrc-1, IRF4, and IL-10. We also performed flow cytometry to detect CD206 expression on CD14+ cells and found that siMCPIP1-Mφs expressed less CD206 than did siNC-Mφs (Fig. 6f). Taken together, the results suggest that MCPIP1 is crucial for Mφs polarization in vivo.

We also explored the role of MCPIP1 in Mφs polarization in vitro. As shown in Fig. 6g, siMCPIP1-Mφs expressed much less CD206 than did siNC-Mφs. In addition, expression of CD206 was upregulated when siNC-Mφs were cocultured with ARP-1 cells, though expression barely changed when siMCPIP1-Mφs were cocultured with ARP-1 cells. These data suggest that Mφs with MCPIP1 knockdown not only display a more M1-like phenotype but that they are also more difficult to be polarized toward the M2-like phenotype by MM cells.

As previous studies have shown that TNF-α, a cytotoxic factor, is involved in the tumoricidal effect of Mφs^19^, we then performed ELISA to detect secretion of TNF-α by Mφs. The results indicated that although siNC-Mφs and siMCPIP1-Mφs secreted little TNF-α, this secretion was upregulated when Mφs were cocultured with RMPI.8226 cells and primary MM cells. In addition, siMCPIP1-Mφ secretion was significantly higher than that of siNC-Mφs when cocultured with MM cells (Fig. 6h). According to Western blot analysis, knocking down MCPIP1 downregulated expression of Arg-1, CD206, and pAMPK and upregulated that of iNOS. In addition, rhCCL2 induced expression of Arg-1, CD206, and pAMPK and suppressed that of iNOS in siNC-Mφs, whereas rhCCL2 had little effect on siMCPIP1-Mφs (Fig. 6i).

All of these in vitro experiments indicate that MCPIP1 is crucial for Mφs polarization.

### CCL2-induced MCPIP1 expression was dependent on the JAK2-STAT3 pathway

**Fig. 7:**
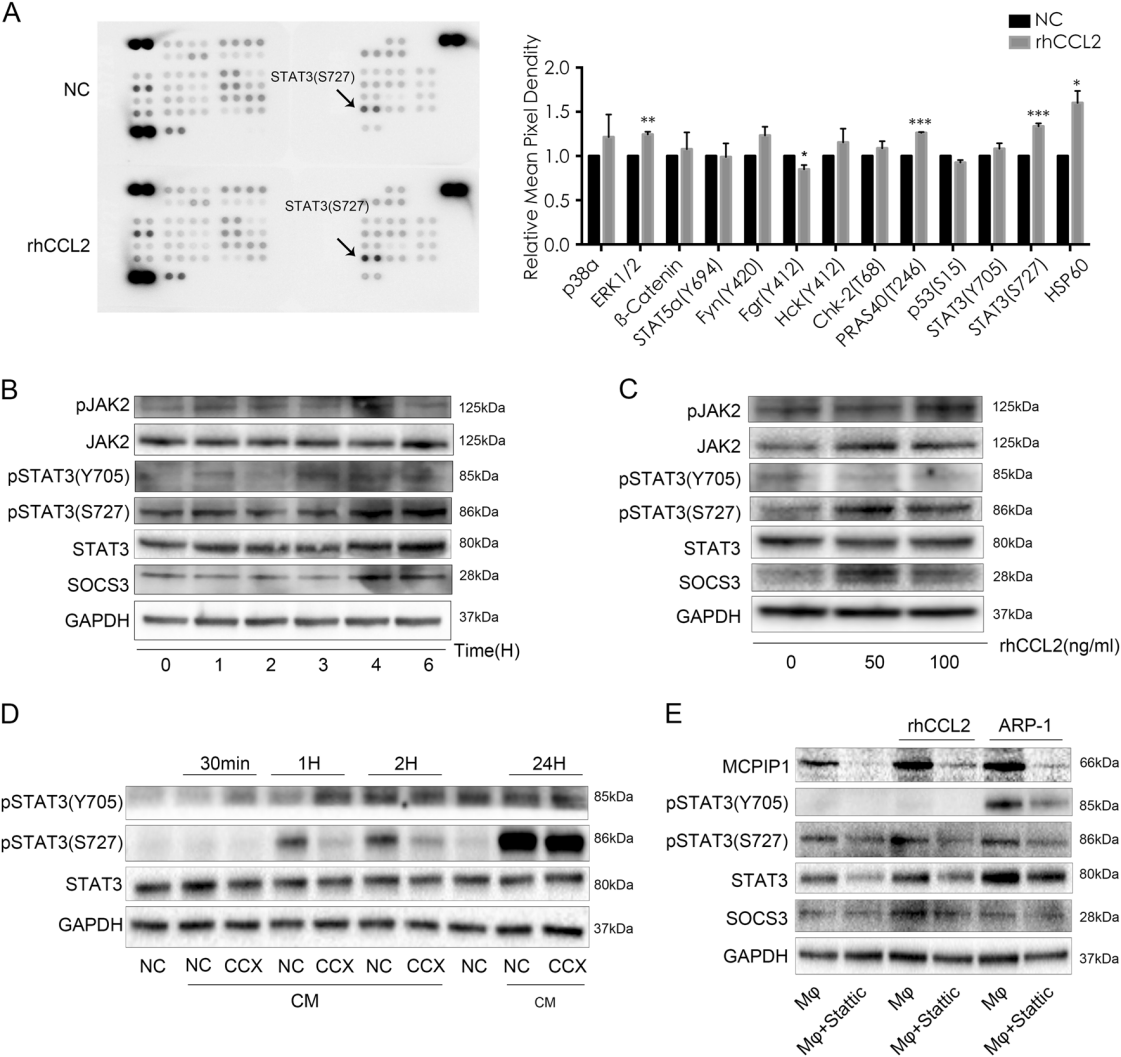
CCL2 induces MCPIP1 expression through the JAK2-STAT3 pathway (**a**) Mφs were treated with or without rhCCL2 (50 ng/mL) for 24 h and then harvested for protein level detection using Proteome Profiler Human Phosphorylation Kinase Antibody Array. Each target was assayed in duplicate. Representative images and quantitative diagrams of the relative content of each protein are expressed as the fold change of the blot intensity normalized to that of the NC group. **b**, **c** Western blotting results for expression of phosphorylated STAT3, JAK2, and SOCS3 in Mφs stimulated with rhCCL2 for the indicated time and concentration. **d** Mφs were exposed to RPMI-8226 conditioned medium (CM) for the indicated time with or without CCX140-B (CCX). Western blotting was applied to detect STAT3 phosphorylation. **e** Mφs were treated with or without the STAT3 inhibitor Stattic (20 µM) for 2 h and then exposed to rhCCL2 (50 ng/mL) or cocultured with ARP-1 cells for 24 h. Western blotting was conducted to determine MCPIP1 expression and phosphorylation of STAT3, STAT3, and SOCS3 in Mφs. Similar results were observed in at least three independent experiments

Finally, we used Proteome Profiler Human Phospho-kinase Antibody Array to determine changes in protein phosphorylation in Mφs caused by rhCCL2. The results showed that CCL2 significantly activated STAT3 by increasing S727 phosphorylation levels in Mφs (Fig. 7a). We next performed Western blotting to confirm this activation, and as shown in Fig. 7b, rhCCL2 resulted in STAT3 phosphorylation at 4 h after exposure, and rhCCL2 also phosphorylated S727-STAT3 in a concentration-dependent manner (Fig. 7c). Furthermore, MM cell conditioned medium activated phosphorylation of STAT3 (S727), and the CCR2 inhibitor CCX140-B inhibited this activation (Fig. 7d). To verify the role of STAT3 in CCL2-induced MCPIP1 expression, we blocked the STAT3 pathway with the STAT3 inhibitor Stattic. As depicted in Fig. 7e, Stattic indeed inhibited STAT3 phosphorylation in Mφs induced by rhCCL2 and ARP-1 cells and significantly downregulated CCL2-induced MCPIP1 protein expression.

Taken together, the results indicate that CCL2 induces MCPIP1 expression via the JAK2-STAT3 signaling pathway in Mφs.

## Discussion

The mechanism of MM chemoresistance is associated with both intrinsic changes in MM cells and the protective efficiency of BMSCs^3^. Mφs, a prominent component in the BM microenvironment of MM, play an important role in protecting MM cells from drug-induced apoptosis.

We previously reported that CCL2 expression was increased in the BM of MM patients and promotes Mφs infiltration into the BM^13^. In this study, we demonstrated that Mφs abundantly express CCL2; we also found that coculture with MM cells further upregulates Mφs’ expression of CCL2. To examine why Mφs upregulated expression of CCL2 when cocultured with MM cells, which is similar to the MM BM microenvironment, we performed mechanism studies to further elucidate the role of increased CCL2 expression in the BM microenvironment.

Interestingly, we found that when newly diagnosed patients received four courses of PCD combined therapy, CCL2 expression in their BM was greatly decreased. According to their clinicopathologic characteristics, almost all of these patients have been in remission due to therapy. Thus, CCL2 expression is tightly related to MM patient treatment status.

**Fig. 8:**
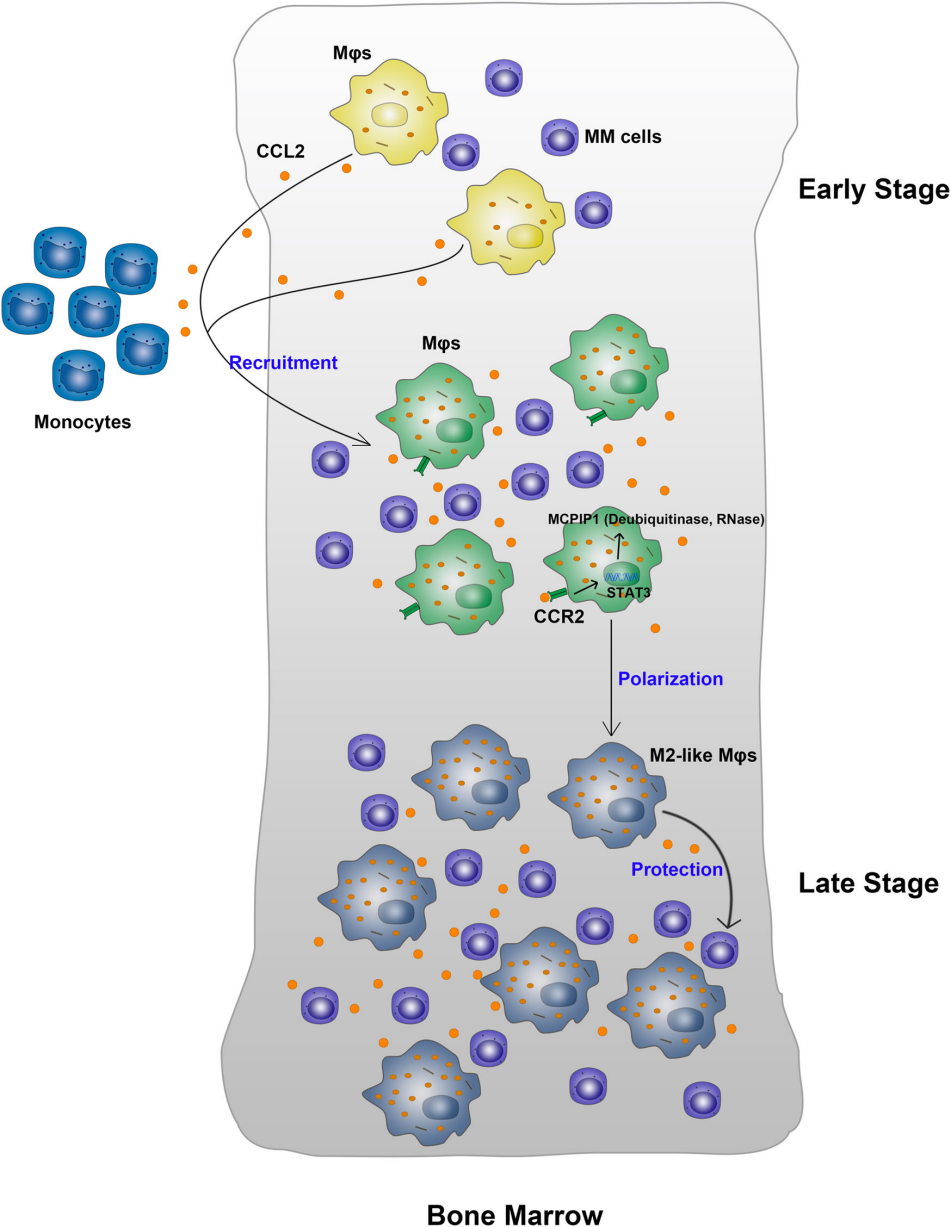
A summarizing figure of main findings presented in the study. CCL2 strongly recruits monocytes to the BM microenvironment. Then these recruited Mφs interact with MM cells and further upregulated Mφs’ expression of CCL2. These increased CCL2-induced MCPIP1 expression in Mφs through STAT3 pathway, polarizing Mφs toward M2-like phenotype and promoting Mφs to protect MM cells from drug-induced apoptosis

CCL2 has long been recognized as a regulator of TAMs in different cancers^29–31^. Some tumor cells also express CCL2, and high-CCL2 expression is associated with tumor cell therapy resistance^32,33^. However, in our study, MM cells barely expressed CCL2, and CCL2 treatment had little effect on MM cells’ survival or proliferation. We showed that CCL2 is important for the protective effect of Mφs on MM cells. In addition, we found that CCL2 could skew the Mφs phenotype toward the M2-like phenotype, which is consistent with the results of some studies in solid tumors^34^. We also found that Mφs with different polarization statuses exert different protective effects on MM cells, which indicates that CCL2 promotes the protective effect of Mφs by polarizing them toward the M2-like phenotype.

Based on mechanistic studies, CCL2 induces expression of MCPIP1 in Mφs. MCPIP1 participates in Mφs polarization and promote the protective capacity of these cells via its dual catalytic activities. We also found that CCL2 induces expression of MCPIP1 via the JAK2-STAT3 signaling pathway. Overall, this study provides the first direct evidence that CCL2 induces Mφs polarization toward the M2-like phenotype and promotes their protective effects on MM cells via MCPIP1.

Interaction of CCL2 with its receptor CCR2 causes signal transduction events that induce the zinc-finger protein MCPIP1, and many studies have revealed that MCPIP1 is a critical immunoregulatory agent. MCPIP1 is essential for preventing aberrant T-cell activation^23^, and it was also reported that MCPIP1 participates in IL4-induced Mφs polarization^35^. Our data show that MCPIP1 indeed enhances the protective effect of Mφs and is crucial for Mφs polarization. Although we found that the protective effect of Mφs is associated with the dual catalytic activities of MCPIP1, the most important functional domain and specific substrate that mediates the Mφs’ effect is not fully defined and requires further study.

Monoclonal antibody-based therapeutics have great promise for many tumors. For example, carlumab, a human IgG1k anti-CCL2 mAb, has shown antitumor activity in preclinical trials; however, another study showed that carlumab was ineffective at yielding long-lasting decreases in serum CCL2 concentrations and thus had minimal clinical effects^36^. Because of the relationship between CCL2 expression and MM patient treatment status, we speculated that CCL2 might act as an effective prognostic factor for MM patients. In addition, new therapeutic strategies targeting MCPIP1 are likely to be promising.

In summary, CCL2 strongly recruits Mφs to the BM microenvironment. These recruited Mφs then interact with MM cells and further upregulate their expression of CCL2. The increased levels of CCL2 in the microenvironment polarize Mφs toward the M2-like phenotype and promote Mφs to protect MM cells from chemotherapy drug-induced apoptosis (Fig. 8). Mechanistically, CCL2 induces expression of MCPIP1, a critical negative regulator of inflammation that mediates Mφs polarization and protection via its dual catalytic activities. This study provides new insight into the mechanism of drug resistance in MM.

## Supplementary information

supplementary Table+Figure

supplementary legends
