# Supplementary material for: CCL2 promotes macrophages-associated chemoresistance via MCPIP1 dual catalytic activities in multiple myeloma
Source: Cell Death Dis. 2019 Oct 14;10(10):781. doi: 10.1038/s41419-019-2012-4 (PMC6791869; doi:10.1038/s41419-019-2012-4)
Supplement: Supplementary file 1 — supplementary Table+Figure [file 41419_2019_2012_MOESM1_ESM.pdf]

Supplementary Table1

Primer sets used in PCR for human are summarized as follows:

**Gene**	**sense**	**antisense**
**GAPDH**	ACGGATTTGGTCGTATTGGGC	TTGACGGTGCCATGGAATTG
**CCL2**	TGTCCCAAAGAAGCTGTGATC	ATTCTTGGGTTGTGGAGTGAG
**ICAM-3**	GGAGTTCCTTTTGCGGGTG	TCAGAGCTGGGACAATCAGTA
**INHAB**	CTCGGAGATCATCACGTTTG	CCTTGGAAATCGAAGTGC
**CD163**	GCTGTGGTAACTTGCATCCTG	GCAGTAGTGTTCCACCCATCA
**Mrc-1**	TCCGGGTGCTGTTCTCCTA	CCAGTCTGTTTTTGATGGCACT
**IL-10**	GACTTTAAGGGTTACCTGGGTTG	TCACATGCGCCTTGATGTCTG
**MCPIP1**	CGCATATGAGTGGCCCCTGTGGAG	CGGGATCCTTACTCACTGGGGTGCTGG
**IRF4**	GCTGATCGACCAGATCGACAG	CGGTTGTAGTCCTGCTTGC
**TNF-α**	CCTCTCTCTAATCAGCCCTCTG	GAGGACCTGGGAGTAGATGAG

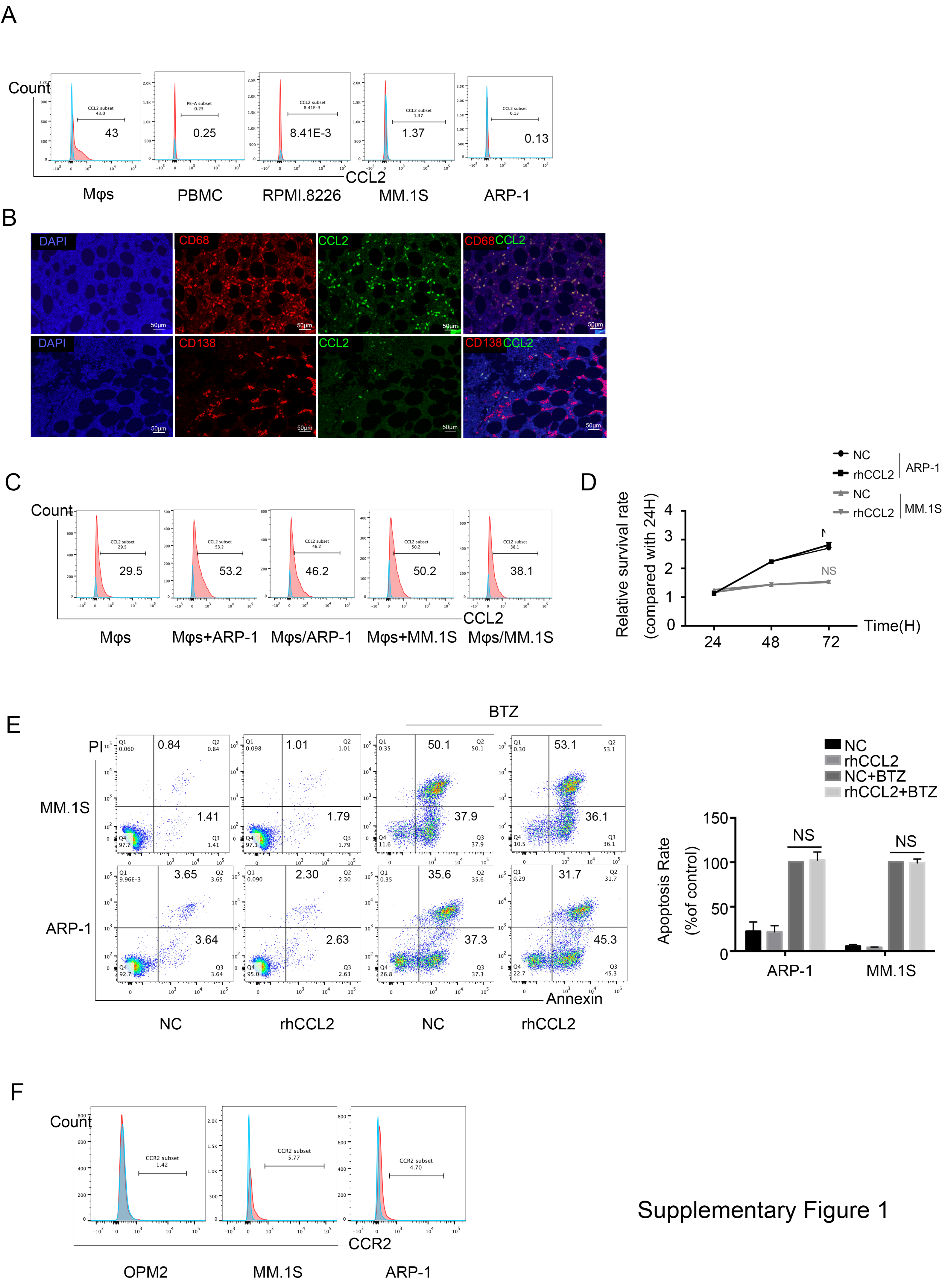

Supplementary Figure 1

A

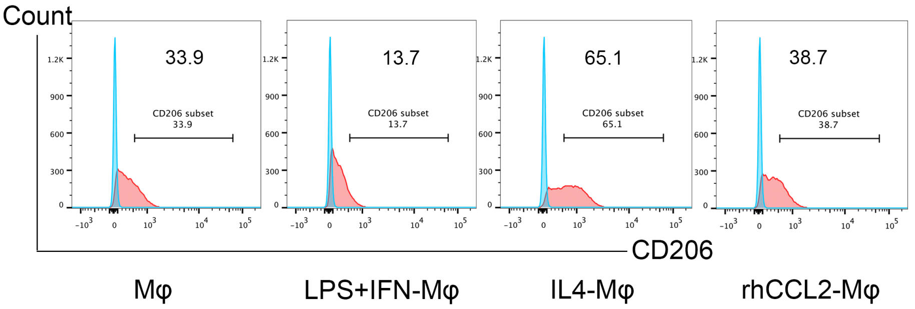

B

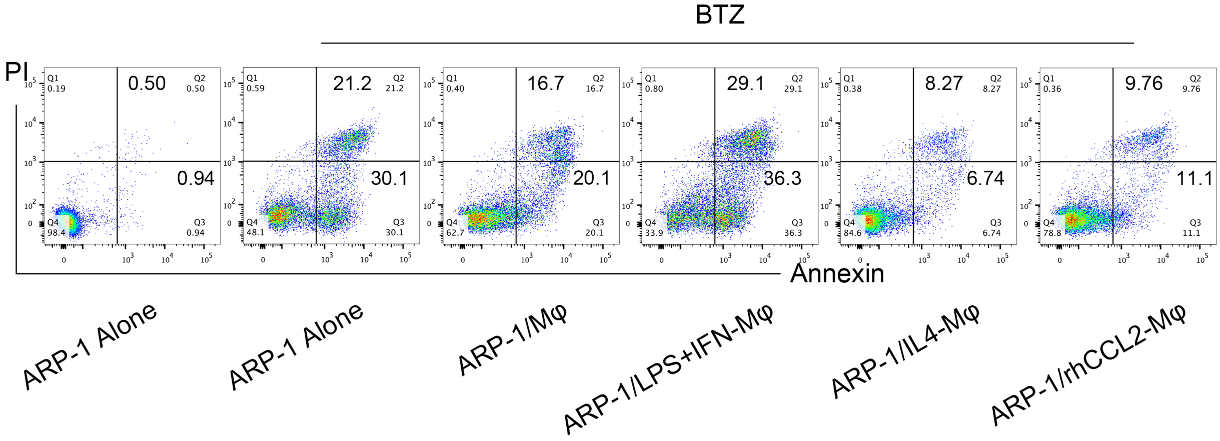

Supplementary Figure 2

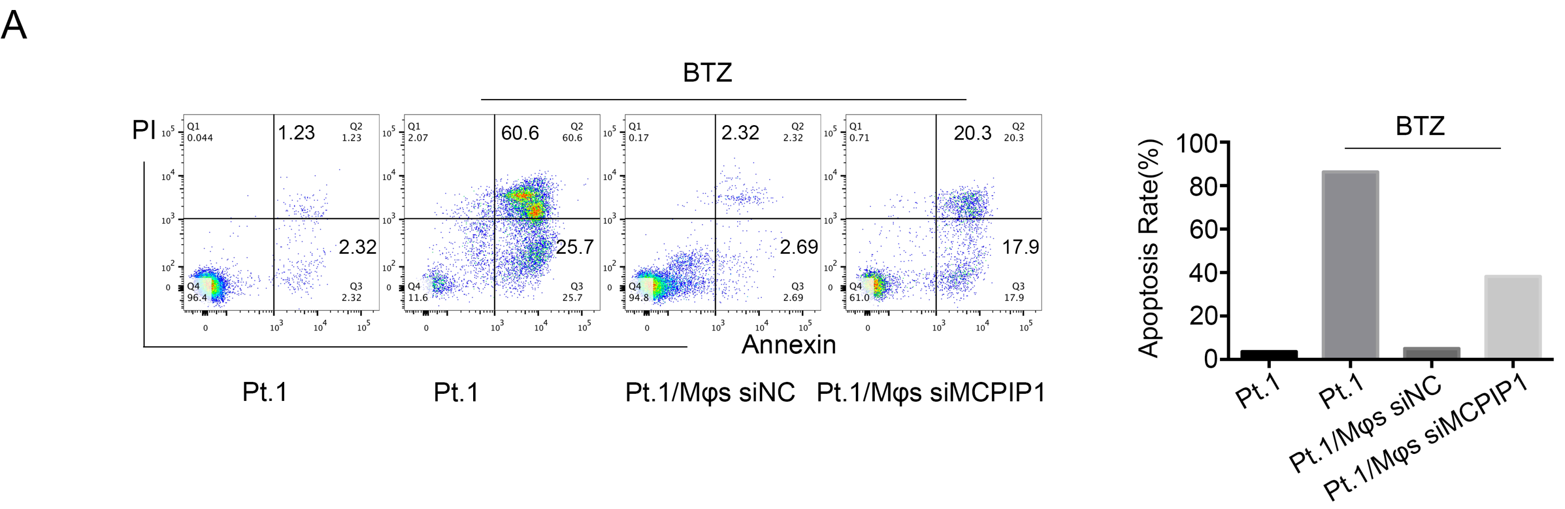

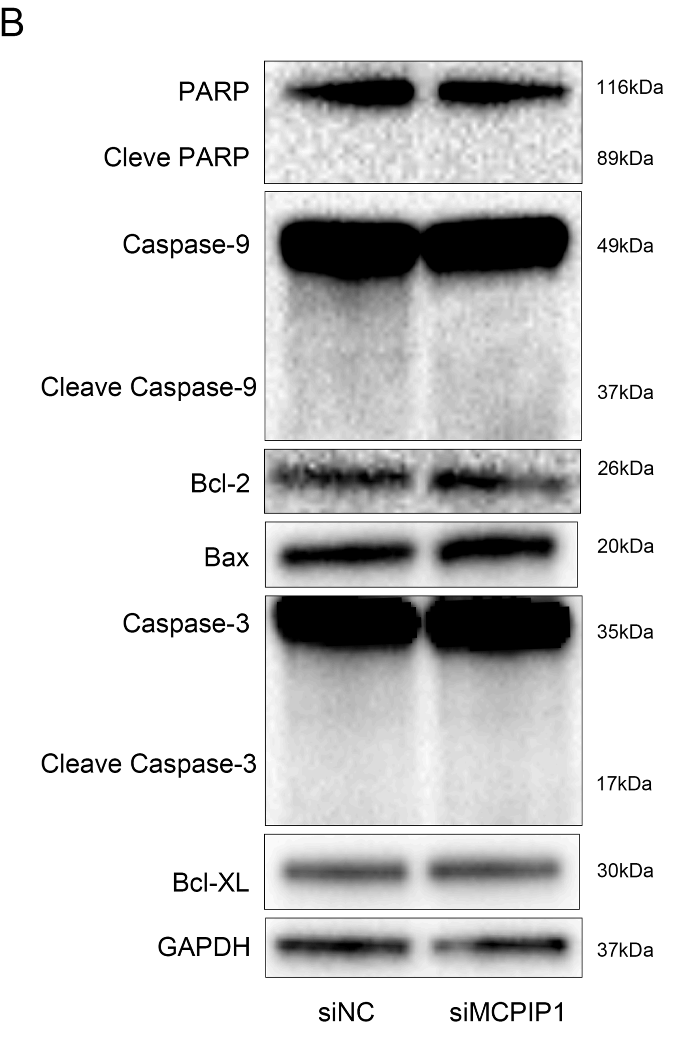

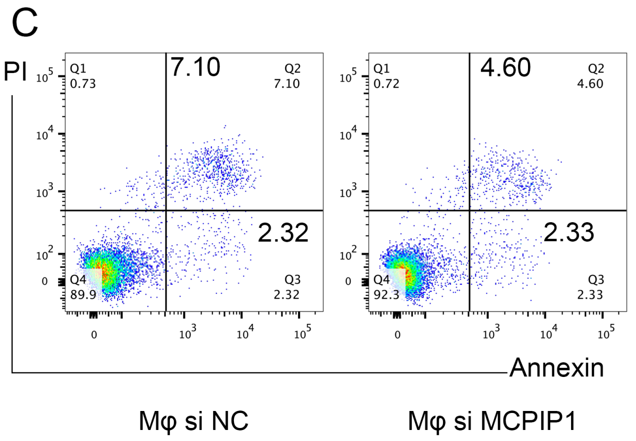

Supplementary Figure 3

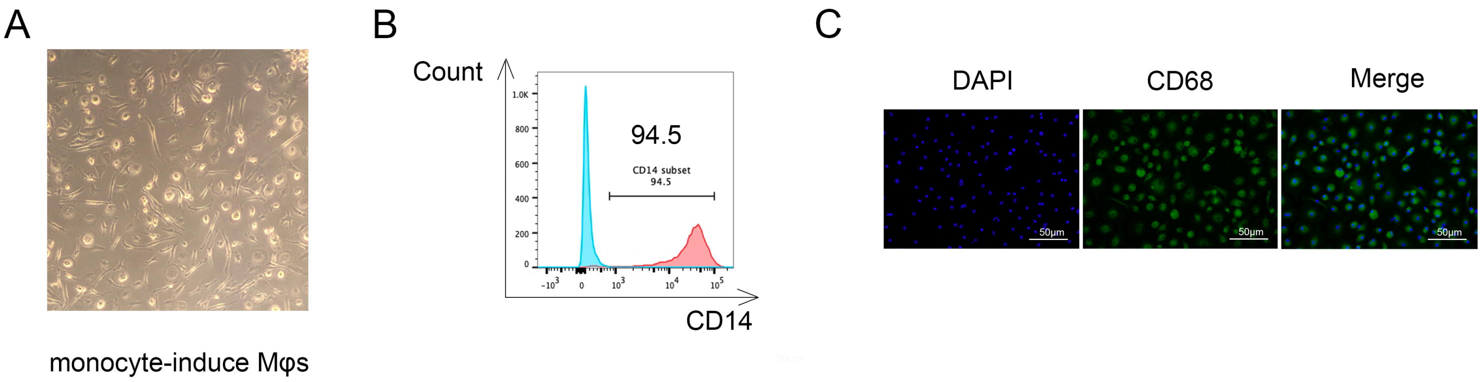

Supplementary Figure 4
