# Supplementary material for: CCL2 promotes macrophages-associated chemoresistance via MCPIP1 dual catalytic activities in multiple myeloma
Source: Cell Death Dis. 2019 Oct 14;10(10):781. doi: 10.1038/s41419-019-2012-4 (PMC6791869; doi:10.1038/s41419-019-2012-4)
Supplement: Supplementary file 2 — supplementary legends [file 41419_2019_2012_MOESM2_ESM.docx]

**Supplementary Figure Legends**

**Supplementary Figure 1**

**CCL2, CCR2 expression of cells and rhCCL2 has little effect in MM cells**

A) Intracellular CCL2 staining assays, the cells were incubated with Leukocyte Activation Cocktail for 4 h and subsequently stained with CCL2 for flow cytometry analysis. The CCL2 expression of Mφs, PBMC and MM cell lines (RPMI.8226, MM.1S, ARP-1). (B) Expression of CCL2 in CD68+ Mφs and CD138+ MM cells from MM patients’ BM biopsies using immunofluorescence. Scale bars, 50 μm. (C) Intracellular CCL2 staining assays, Mφs were cultured alone (Mφs), or cocultured with MM cells directly (Mφs+ARP-1, Mφs+MM.1S), or through transwell chambers (Mφs/ARP-1, Mφs/MM.1S) for 24h. The CCL2 expression of Mφs.(D) MM cells (ARP-1, MM.1S) were treated with or without rhCCL2 (50 ng/ml) for the indicated times. Cell growth was assessed using a CCK-8 proliferation assay. All absorbance values were normalized to those of untreated (control) samples at the 24 h time point. Values are presented as means ± SD. (E) Representative flow cytometry analysis showed the apoptosis rate of ARP-1 and MM.1S cells induced by bortezomib (BTZ) in the absence or presence of rhCCL2 (50 ng/ml) for 24 h. Summarized data from three independent experiments are shown. Values are presented as means ± SD.(F) CCR2 expression of MM cells (OPM2, MM.1S and ARP-1) were detected by flow cytometry. NS: not significant.

**Supplementary Figure 2**

**Differently polarized Mφs have different effects on MM drug resistance.**

A) Mφs were exposed of LPS (100ng/ml) +IFNγ(20ng/ml), IL-4 (20 ng/ml), rhCCL2(50ng/ml) for 24h, followed by flow cytometry to detect CD206 expression in Mφs. B) Percentage of bortezomib (BTZ, 10 nM)-induced apoptotic ARP-1 cells upon direct coculture with differently treated Mφs (Mφs, LPS+IFNγ-Mφs, IL4-Mφs and rhCCL2-Mφs). Similar results were observed in at least three independent experiments.

**Supplementary Figure 3**

**Knocking down MCPIP1 hindered Mφs from protecting primary myeloma cells and had little effect on Mφs viability.**

A) Percentage of bortezomib (BTZ, 10 nM)-induced apoptotic primary MM cells from a patient (Pt.1) in different culture conditions: culture alone (Pt.1), coculture with Mφs transfected with a scrambled nontargeting siRNA (Pt.1/Mφs siNC), and coculture with Mφs transfected with MCPIP1 specific siRNA (Pt.1/Mφs siMCPIP1). A representative flow cytometry analysis and quantitative results showed the apoptosis of primary MM cells induced by BTZ. (B) Mφs were transfected with a scrambled nontargeting siRNA or MCPIP1 specific siRNA (siNC, siMCPIP1). Immunoblot analysis were conducted to detect the expression of apoptosis-related proteins in Mφs.(C) A representative flow cytometry analysis showed the apoptosis of Mφs which were transfected with different siRNA. Similar results were observed in at least three independent experiments.

**Supplementary Figure 4**

**The morphological and phenotypical characterization of macrophages.**

Mφs were differentiated from PBMCs in medium containing M-CSF (20 ng/ml) for 7 days. A) Mφs had a spindle-like morphology. Inverted phase contrast microscope 100×. B) The expression of CD14 in Mφs were detected by flow cytometry analysis. C) The expression of CD68 in Mφs were detected by immunofluorescence.
